# Supplementary material for: Atomic and electronic modulation of self-supported nickel-vanadium layered double hydroxide to accelerate water splitting kinetics
Source: Nat Commun. 2019 Aug 29;10:3899. doi: 10.1038/s41467-019-11765-x (PMC6715676; doi:10.1038/s41467-019-11765-x)
Supplement: Supplementary file 1 — Supporting information [file 41467_2019_11765_MOESM1_ESM.pdf]

Electronic Supplementary Information

Atomic and electronic modulation of self-supported nickel-vanadium layered double hydroxide to accelerate water splitting kinetics

Wang et al.

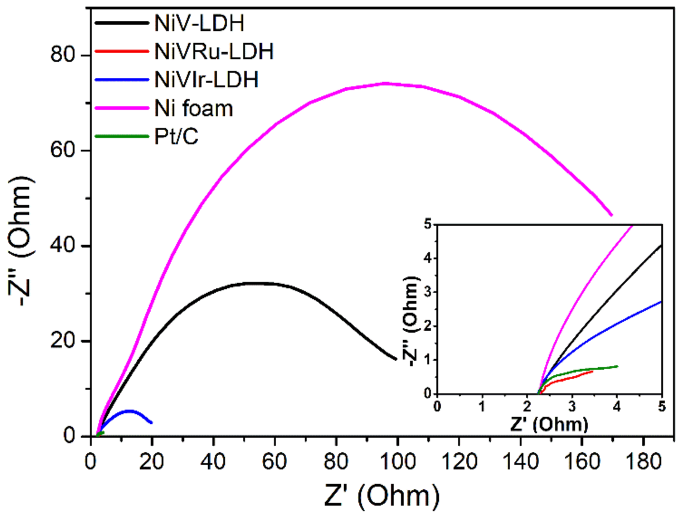

Supplementary Fig. 1 Nyquist plots of the electrocatalysts.

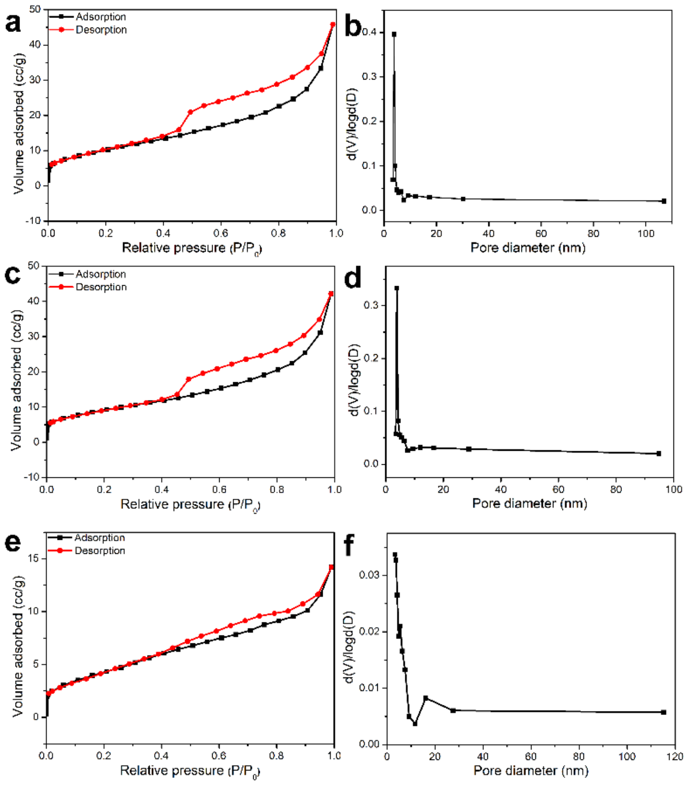

Supplementary Fig. 2 BET surface areas and pore size distributions. Nitrogen adsorption-desorption isotherm and the corresponding pore size distribution of (**a**, **b**) NiV-LDH, (**c**, **d**) NiVRu-LDH and (**e**, **f**) NiVIr-LDH, respectively.

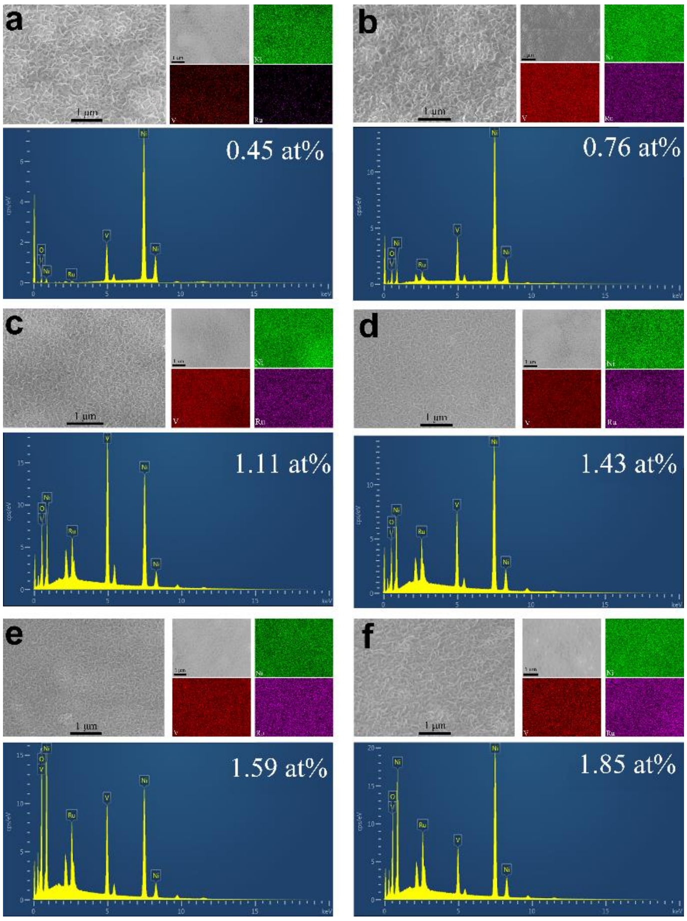

Supplementary Fig. 3 SEM images, mapping images and EDS spectra. the corresponding mapping images of Ni, V and Ru elements and the EDS spectra of the NiVRu-LDH with different Ru content. The Ru in NiVRu-LDH is 0.45 (**a**), 0.76 (**b**), 1.11 (**c**), 1.43 (**d**), 1.59 (**e**) and 1.85 at% (**f**), respectively.

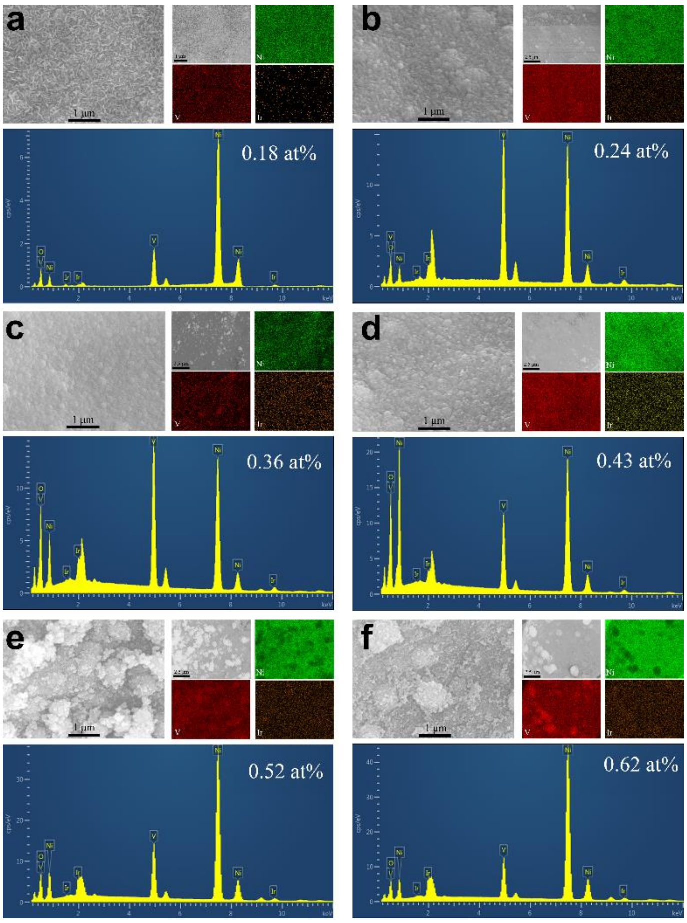

Supplementary Fig. 4 SEM images, mapping images and EDS spectra. SEM images, the corresponding mapping images of Ni, V and Ir elements and the EDS spectra of the NiVIr-LDH with different Ir content. The Ir in NiVIr-LDH is 0.18 (**a**), 0.24 (**b**), 0.36 (**c**), 0.43 (**d**), 0.52 (**e**) and 0.62 at% (**f**), respectively.

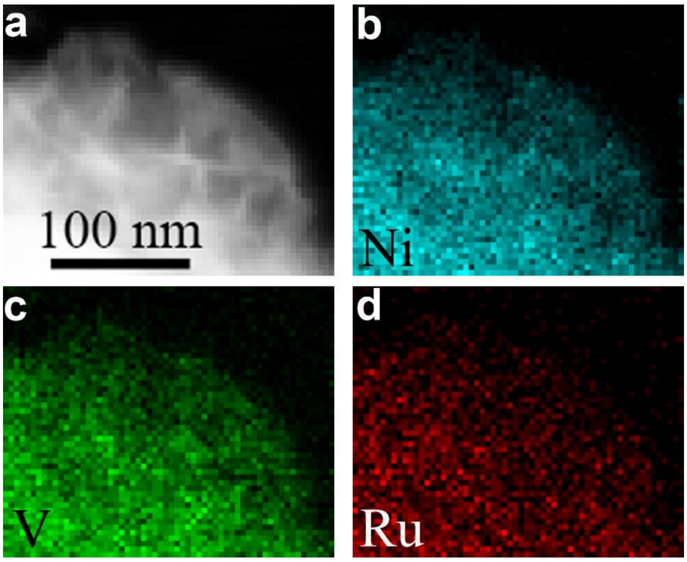

Supplementary Fig. 5 STEM and EDS mapping images. (**a**) STEM image and the corresponding EDS mapping images for (**b**) Ni, (**c**) V and (**d**) Ru of NiVRu-LDH.

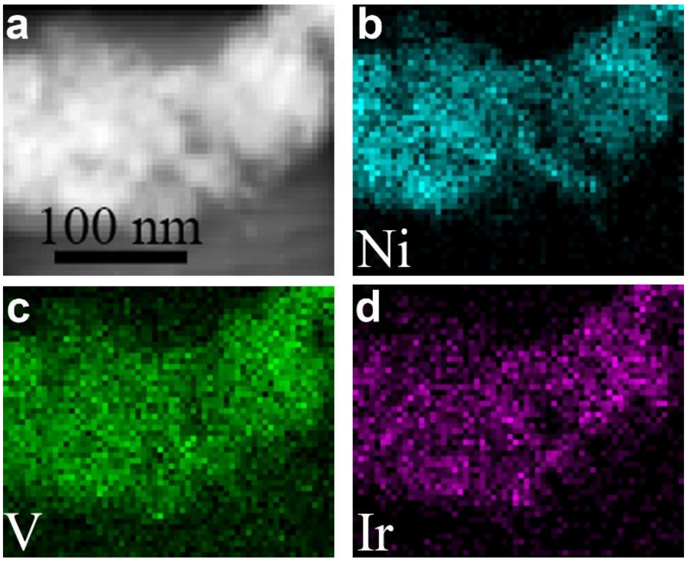

Supplementary Fig. 6 STEM and EDS mapping images. (**a**) STEM image and the corresponding EDS mapping images for (**b**) Ni, (**c**) V and (**d**) Ir of NiVIr-LDH.

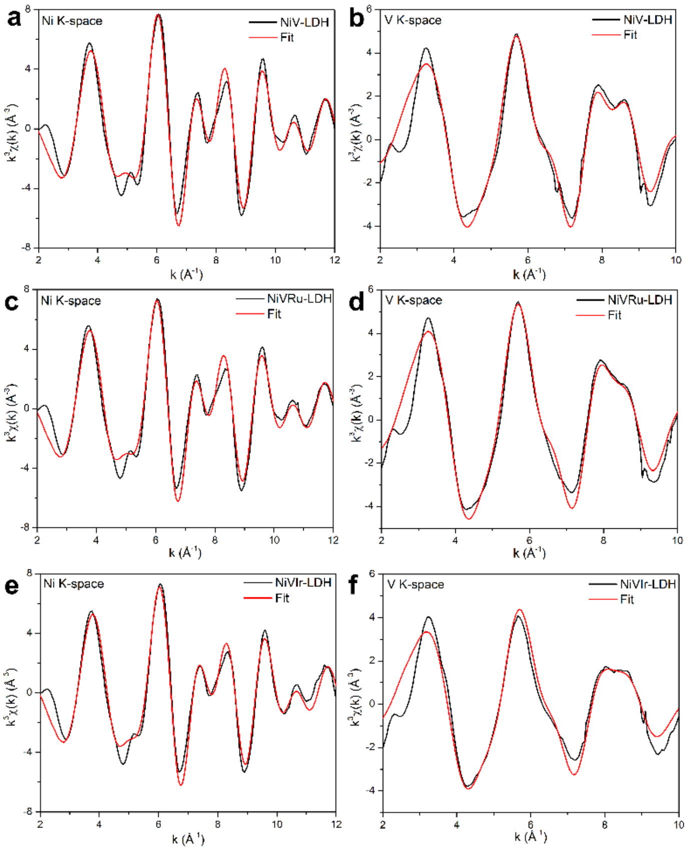

Supplementary Fig. 7 XANES spectra. Ni K-edge extended XANES oscillation functions $k^3\chi(k)$ of (**a**) NiV-LDH, (**c**) NiVRu-LDH and (**e**) NiVIr-LDH. V K-edge extended XANES oscillation functions $k^3\chi(k)$ of (**b**) NiV-LDH, (**d**) NiVRu-LDH and (**f**) NiVIr-LDH.

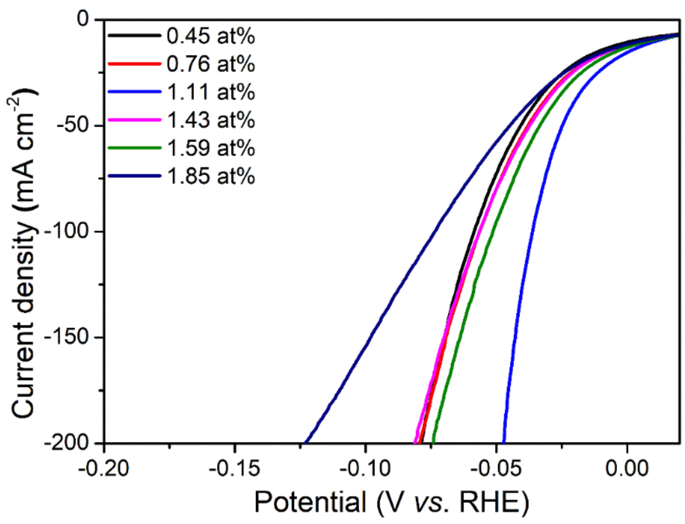

Supplementary Fig. 8 Polarization curves for HER. The HER polarization curves of NiVRu-LDH with different Ru contents.

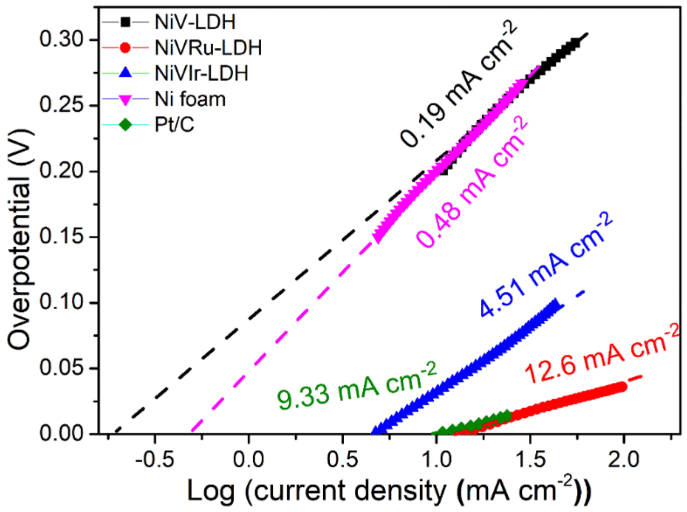

Supplementary Fig. 9 The exchange current densities. The exchange current densities of NiV-LDH, NiVRu-LDH, NiVIr-LDH, Ni foam and Pt/C.

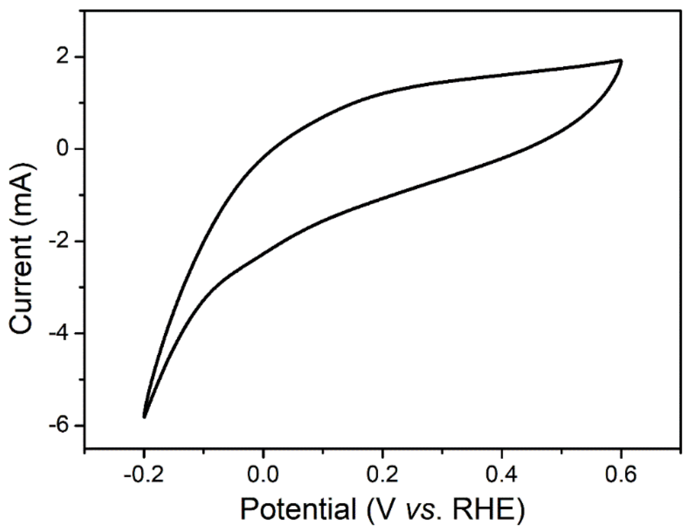

Supplementary Fig. 10 CV curves. CV curves for NiVRu-LDH recorded between -0.2 V and 0.6 V *vs.* RHE in 1.0 M PBS (pH=7) at a scan rate of 50 mV s^{-1}.

Since the difficulty in attributing the observed peaks to a given redox couple, the number of active sites should be proportional to the integrated charge over the CV curve. Assuming a one-electron process for both reduction and oxidation, the upper limit of active sites (n) for NiVRu-LDH could be calculated according to the follow equation:

$$n = Q/2F \tag{1}$$

where F=96485.3 C/mol and Q are the Faraday constant and the whole charge of CV curve, respectively. By this equation and the CV curves, taking NiVRu-LDH as an example, the detailed calculation process of n can be provided as follows:

$$Q = \frac{\int VA}{v} = \frac{0.00544}{0.05} = 0.1088\ C \tag{2}$$

$$n = \frac{Q}{2F} = \frac{0.1088}{2\times 96485.3} = 5.64\times 10^{-7}\ mol \tag{3}$$

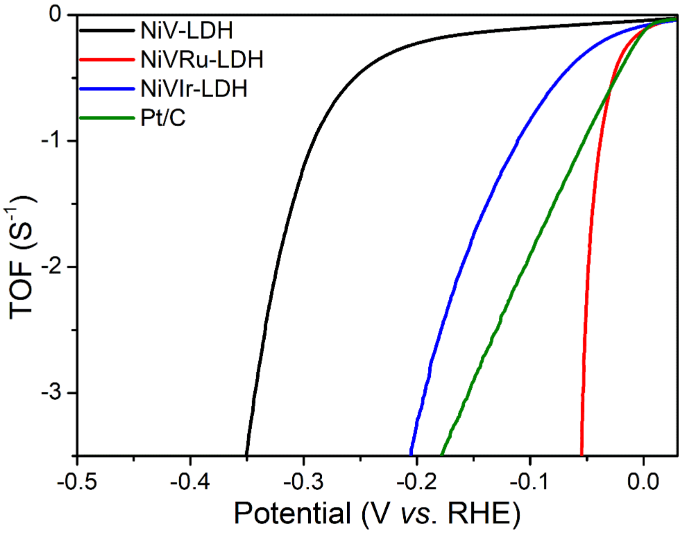

Supplementary Fig. 11 Calculated TOFs. The calculated TOFs of NiV-LDH, NiVRu-LDH, NiVIr-LDH and Pt/C.

Assuming that all of active sites were entirely accessible to the electrolyte, the TOF values were calculated and plotted against the potential. The following formula was used to calculate TOF:

$$TOF=I/2nF \tag{4}$$

where F and n are the Faraday constant and the number of active sites, respectively; I is the current density of LSV curve.

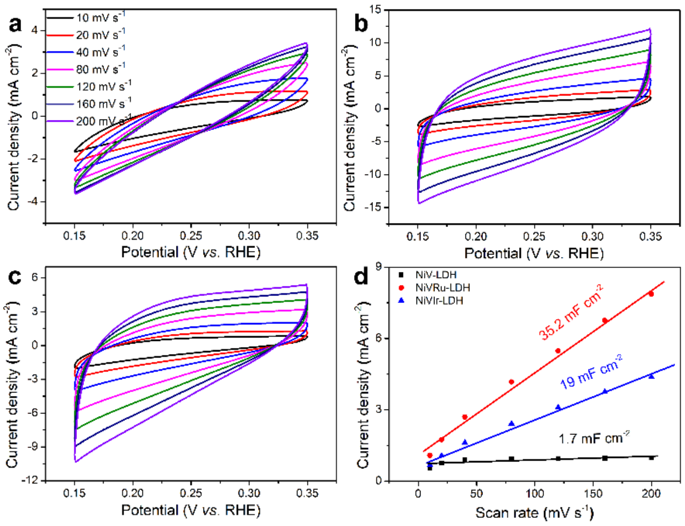

Supplementary Fig. 12 CVs and the corresponding capacitive currents. CVs for (**a**) NiV-LDH, (**b**) NiVRu-LDH and (**c**) NiVIr-LDH at different scan rates. (**d**) The corresponding capacitive currents at 0.25 V as a function of scan rate for NiV-LDH, NiVRu-LDH and NiVIr-LDH.

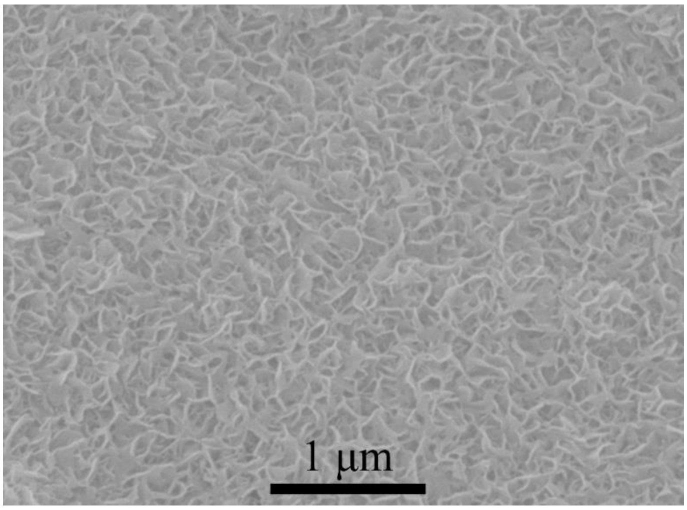

Supplementary Fig. 13 SEM image. SEM image of the NiVRu-LDH after a long time HER stability test.

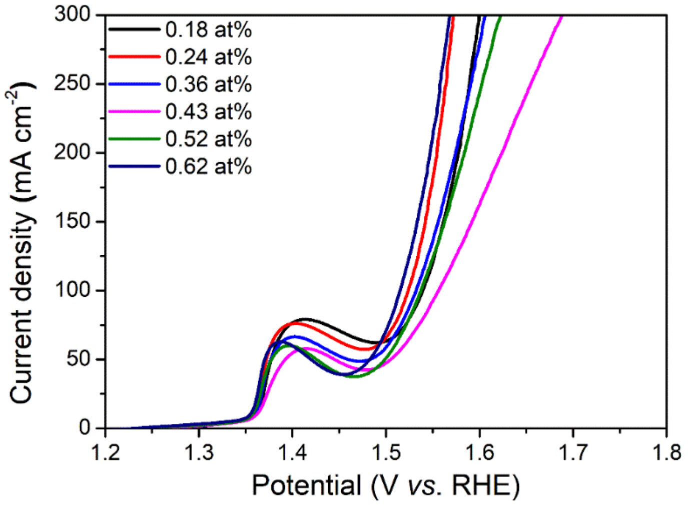

Supplementary Fig. 14 Polarization curves for OER. The OER polarization curves of NiVIr-LDH with different Ir contents.

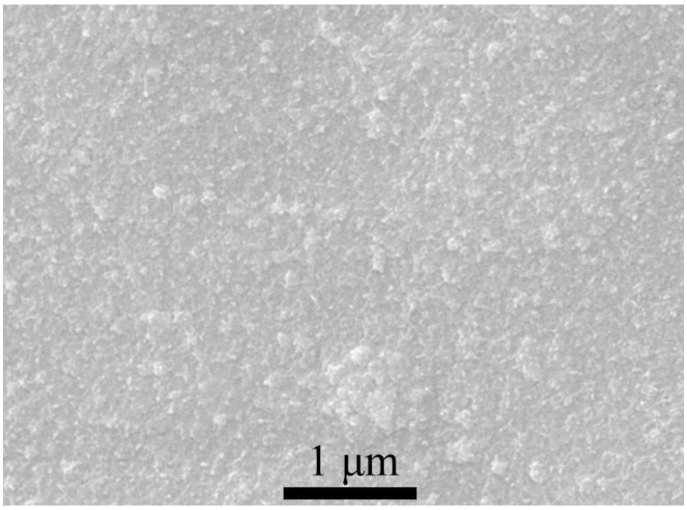

Supplementary Fig. 15 SEM images. SEM image of the NiVIr-LDH after a long time OER stability test.

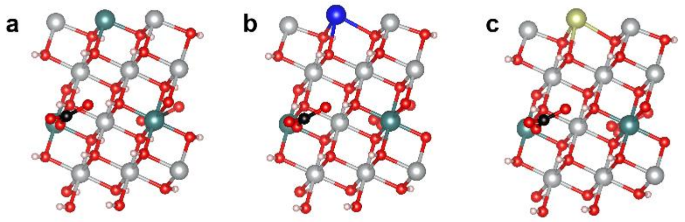

Supplementary Fig. 16 Structural modes. The as-built structural models of (**a**) the NiV-LDH, (**b**) NiVRu-LDH and (**c**) the NiVIr-LDH.

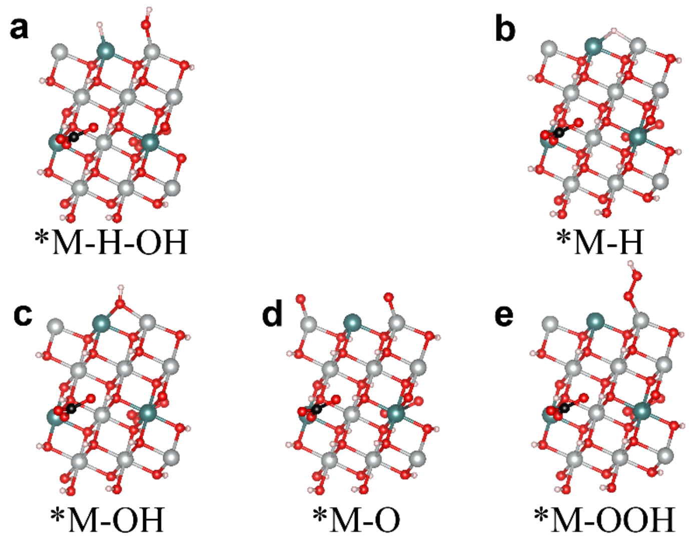

Supplementary Fig. 17 Structural modes. The as-built structural models of the NiV-LDH for different steps of HER (**a**) and (**b**), and of OER (**c**), (**d**) and (**e**).

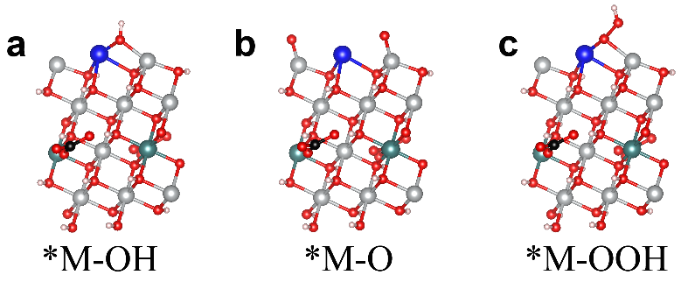

Supplementary Fig. 18 Structural modes. The as-built structural models of the NiVRu-LDH for different steps of OER (**a**), (**b**) and (**c**).

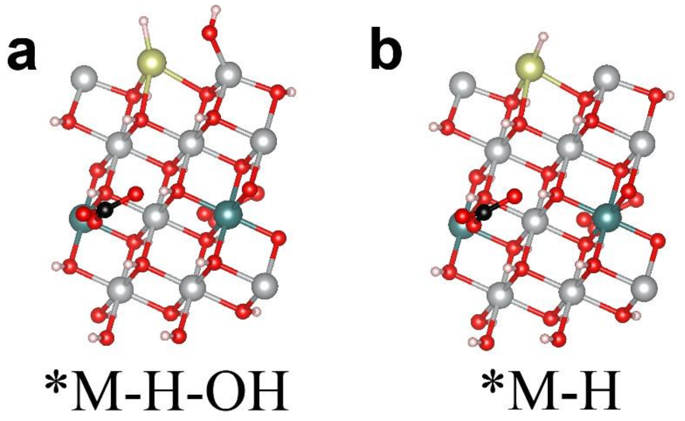

Supplementary Fig. 19 Structural modes. The as-built structural models of the NiVIr-LDH for different steps of HER (**a**) and (**b**).

Supplementary Table 1. Summary the fitting parameters of Ni and V *K*-edge EXFAS curves for the as-prepared NiV-LDH, NiVRu-LDH and NiVIr-LDH catalysts.

Sample	Path	C.N.	R (Å)	$\sigma^2 \times 10^3$ (Å^2)	ΔE (eV)	R factor
NiV-LDH	Ni-O	6.4±0.6	2.04±0.01	8.2±0.8	-6.4±1.1	0.005
	Ni-Ni/V	5.1±0.7	3.09±0.01	9.6±1.0	0.4±1.3	
NiVRu-LDH	Ni-O	6.5±0.5	2.03±0.01	8.4±0.8	-6.6±1.0	0.004
	Ni-Ni/V	4.6±0.6	3.08±0.01	9.8±1.0	0.8±1.3	
NiVIr-LDH	Ni-O	6.5±0.6	2.03±0.01	8.2±0.9	-6.1±1.2	0.006
	Ni-Ni/V	4.2±0.8	3.08±0.01	9.5±1.3	0.8±1.7	
NiV-LDH	V-O	5.7±0.6	1.68±0.01	6.7±0.9	1.8±1.6	0.008
	V-Ni/V	5.3±2.5	3.40±0.03	12.3±3.8	5.6±0.6	
NiVRu-LDH	V-O	6.5±0.6	1.68±0.01	7.1±0.9	2.5±1.5	0.008
	V-Ni/V	3.8±2.3	3.37±0.04	17.2±5.6	3.7±4.8	
NiVIr-LDH	V-O	6.4±0.9	1.66±0.01	8.7±1.3	-2.2±2.3	0.014
	V-Ni/V	2.8±2.5	3.37±0.05	18.6±9.4	2.5±6.8	

Note: ΔE, inner potential correction; σ^2, Debye Waller factor to account for both thermal and structural disorders; *R*-factor, indicating the goodness of the fit.

The obtained XAFS data was processed in Athena (version 0.9.25) for background, pre-edge line and post-edge line calibrations. Then Fourier transformed fitting was carried out in Artemis (version 0.9.25). The k^3 weighting, k-range of 3-13 Å^{-1} and R range of 1-3 Å were used for 2 shell fitting. The model of bulk Ni and NiV-LDH were used to calculate the simulated scattering paths. The four parameters, coordination number, bond length, Debye-Waller factor and E_0 shift (CN, R, σ^2, ΔE_0) were fitted without anyone was fixed, constrained, or correlated.

For Wavelet Transform analysis, the χ(k) exported from Athena was imported into the Hama Fortran code. The parameters were listed as follow: R range, 1 - 4 Å, k range, 0 - 13 $Å^{-1}$; k weight, 3; and Morlet function with κ=10, σ=1 was used as the mother wavelet to provide the overall distribution.

Supplementary Table 2. Comparison of HER performances for NiVRu-LDH with other selected electrocatalysts.

Electrocatalysts	Electrolyte	Overpotential (mV)/ *j* mA/cm^2	Tafel solpe (mV dec^{-1})	TOF (S^{-1})	Ref.
NiVRu-LDH	1 M KOH	12/10<br>38/100<br>48/200	40	2.2<br>(50 mV)	This work
$MoNi_4/MoO_2$@Ni	1 M KOH	15/10	30	N/A	1
$NiCo_2P_x$	1 M KOH	58/10	34.3	0.056 (100 mV)	2
Ni–MoO_2-450 NWs/CC	1 M KOH	40/10	30	N/A	3
NC/NiMo/$NiMoO_x$	1 M KOH	29/10	46	N/A	4
RuCoP	1 M KOH	20/38	37	7.26<br>(100 mV)	5
Ru-MoO_2	1 M KOH	29/10	31	N/A	6
IrW/C	0.1 M KOH	29/10	64	1.95<br>(10 mV)	7
Ru@C_2N	1 M KOH	17/10	38	1.66<br>(50 mV)	8
Ru/C_3N_4/C	0.1 M KOH	79/10	N/A	4.2<br>(100 mV)	9

Supplementary Table 3. Comparison of OER performances for NiVIr-LDH with other selected electrocatalysts.

Electrocatalysts	Electrolyte	Overpotential (mV)	*j* mA/cm^2	Ref.
NiVIr-LDH	1 M KOH	180<br>243<br>247	10<br>50<br>100	This work
NiV LDHs	1 M KOH	310	10	10
IrO_2 Nanoneedles	1 M H_2SO_4	313	10	11
IrO_2/CNT	0.5 M H_2SO_4	293	10	12
NiFeMn LDHs	1 M KOH	289	20	13
Ir_3Cu MAs	0.1 M $HClO_4$	298	10	14
IrOOH nanosheets	0.1 M $HClO_4$	344	10	15
IrW/C	0.1 M $HClO_4$	300	8.1	7
$IrCo_{0.65}$ NDs	0.1 M $HClO_4$	281	10	16
Ir/g-C_3N_4/NG	0.5 M H_2SO_4	287	10	17

Supplementary Table 4. Comparison of catalysts for overall water splitting performances for NiVIr-LDH||NiVRu-LDH with other electrocatalysts.

Electrode pair	Electrolyte	Potential (V) at 10 mA cm^{-2}	Ref.
NiVIr-LDH||NiVRu-LDH	1 M KOH	1.42	This work
FeP/Ni_2P	1 M KOH	1.42	18
Ni_2P-NiP_2 HNPs||NiFe-LDH	1 M KOH	1.48	19
$Ni_{0.7}Fe_{0.3}PS_3$@MXene||$Ni_{0.7}Fe_{0.3}PS_3$@MXene	1 M KOH	1.65	20
N-Ni_3S_2/NF||N-Ni_3S_2/NF	1 M KOH	1.48	21
Co_3O_4-MTA||Co_3O_4-MTA	1 M KOH	1.63	22
VOOH||VOOH	1 M KOH	1.62	23
Cu@NiFe LDH || Cu@NiFe LDH	1 M KOH	1.54	24
MoS_2/Ni_3S_2	1 M KOH	1.56	25
Ni/Ni_8P_3	1 M KOH	1.61	26

Supplementary Table 5. The correction of zero point energy and entropy of the adsorbed and gaseous species.

	ZPE (eV)	TS (eV)
*OOH	0.35	0
*O	0.05	0
*OH	0.31	0.01
*H	0.18	0.03
H_2O	0.56	0.67
H_2	0.27	0.41

Supplementary Note 1: Role of urea in LDH synthesis.

To predict the behavior of LDHs in the applications, the control and reproducibility of their crystal and particle properties is important and a high crystallinity is necessary. The urea hydrolysis method introduced by Costantino et al. was an important advancement in this regard[27, 28]. The urea method utilizes urea instead of NaOH as the precipitating agent. The advantage of using urea is that the urea hydrolysis progresses slowly which leads to a low degree of super saturation during precipitation. Urea is a weak Bronsted base (pK_b = 13.8). It is highly soluble in water and its controlled hydrolysis in aqueous solutions can yield ammonium cyanate or its ionic form (NH_4^+, NCO^-). Prolonged hydrolysis results in either CO_2 in an acidic medium or CO_3^{-2} in a basic environment as shown below[29, 30, 31]:

$$NH_2 - CO - H_2N \rightarrow NH_4^+ + NCO^- \tag{5}$$

$$NCO^- + 2H_2O \rightarrow NH_4^+ + CO_3^{-2} \tag{6}$$

$$NCO^- + 2H^+ + 2H_2O \rightarrow NH_4^+ + HCO_3^- \tag{7}$$

A reaction temperature above 60 °C produces the progressive decomposition of urea in ammonium hydroxide leading to a homogeneous precipitation. This method has been already employed for the synthesis of well crystallized MAl-LDH (M = Li, Mg, Ni, Co), NiFe -LDH, CoTi -LDH and even three-component LDH with large particle sizes[32, 33, 34].

Supplementary references

1. Zhang J, *et al.* Efficient hydrogen production on $MoNi_4$ electrocatalysts with fast water dissociation kinetics. *Nat. Commun.* **8**, 15437 (2017).
2. Zhang R, *et al.* Ternary $NiCo_2P_x$ Nanowires as pH-Universal Electrocatalysts for Highly Efficient Hydrogen Evolution Reaction. *Adv. Mater.* **29**, 1605502 (2017).
3. Ren B, Li D, Jin Q, Cui H, Wang C. Integrated 3D self-supported Ni decorated MoO_2 nanowires as highly efficient electrocatalysts for ultra-highly stable and large-current-density hydrogen evolution. *J. Mater. Chem.* **5**, 24453-24461 (2017).
4. Hou J, Wu Y, Cao S, Sun Y, Sun L. Active Sites Intercalated Ultrathin Carbon Sheath on Nanowire Arrays as Integrated Core–Shell Architecture: Highly Efficient and Durable Electrocatalysts for Overall Water Splitting. *Small* **13**, 1702018 (2017).
5. Xu J, *et al.* Boosting the hydrogen evolution performance of ruthenium clusters through synergistic coupling with cobalt phosphide. *Energ. Environ. Sci.* **11**, 1819-1827 (2018).
6. Jiang P, *et al.* Pt-like electrocatalytic behavior of Ru–MoO_2 nanocomposites for the hydrogen evolution reaction. *J. Mater. Chem. A* **5**, 5475-5485 (2017).
7. Lv F, *et al.* Iridium–Tungsten Alloy Nanodendrites as pH-Universal Water-Splitting Electrocatalysts. *ACS cent. Sci.* **4**, 1244-1252 (2018).
8. Mahmood J, *et al.* An efficient and pH-universal ruthenium-based catalyst for the hydrogen evolution reaction. *Nat. Nanotech.* **12**, 441-446 (2017).
9. Zheng Y, *et al.* High Electrocatalytic Hydrogen Evolution Activity of an Anomalous Ruthenium Catalyst. *J. Am. Chem. Soc.* **138**, 16174-16181 (2016).
10. Fan K, *et al.* Nickel–vanadium monolayer double hydroxide for efficient electrochemical water oxidation. *Nat. Commun.* **7**, 11981-11981 (2016).
11. Lim J, *et al.* Ultrathin IrO_2 Nanoneedles for Electrochemical Water Oxidation. *Adv. Fun. Mater.* **28**, 1704796 (2018).
12. Guan J, Li D, Si R, Miao S, Zhang F, Li C. Synthesis and Demonstration of Subnanometric Iridium Oxide as Highly Efficient and Robust Water Oxidation Catalyst. *ACS Catal.* **7**, 5983-5986 (2017).
13. Lu Z, Qian L, Tian Y, Li Y, Sun X, Duan X. Ternary NiFeMn layered double hydroxides as highly-efficient oxygen evolution catalysts. *Chem. Commun.* **52**, 908-911 (2016).
14. Shi Q, *et al.* Nanovoid Incorporated Ir_xCu Metallic Aerogels for Oxygen Evolution Reaction Catalysis. *ACS Energy Lett.* **3**, 2038-2044 (2018).
15. Weber D, *et al.* IrOOH nanosheets as acid stable electrocatalysts for the oxygen evolution reaction. *J. Mater. Chem.* **6**, 21558-21566 (2018).
16. Fu L, Zeng X, Cheng G, Luo W. IrCo Nanodendrite as an Efficient Bifunctional Electrocatalyst for Overall Water Splitting under Acidic Conditions. *ACS Appl. Mater. Inter.* **10**, 24993-24998 (2018).

17. Jiang B, Wang T, Cheng Y, Liao F, Wu K, Shao M. Ir/g-C_3N_4/Nitrogen-Doped Graphene Nanocomposites as Bifunctional Electrocatalysts for Overall Water Splitting in Acidic Electrolytes. *ACS Appl. Mater. Inter.* **10**, 39161-39167 (2018).
18. Yu F, *et al.* High-performance bifunctional porous non-noble metal phosphide catalyst for overall water splitting. *Nat. Commun.* **9**, 2551 (2018).
19. Liu T, Li A, Wang C, Zhou W, Liu S, Guo L. Interfacial Electron Transfer of Ni_2P–NiP_2 Polymorphs Inducing Enhanced Electrochemical Properties. *Adv. Mater.* **30**, 1803590 (2018).
20. Du C, *et al.* Self‐Assemble and In Situ Formation of $Ni_{1-x}Fe_xPS_3$ Nanomosaic‐Decorated MXene Hybrids for Overall Water Splitting. *Adv. Energy Mater.* **8**, 1801127 (2018).
21. Chen P, *et al.* 3D Nitrogen-Anion-Decorated Nickel Sulfides for Highly Efficient Overall Water Splitting. *Adv. Mater.* **29**, 1701584 (2017).
22. Zhu YP, Ma T, Jaroniec M, Qiao S. Self‐Templating Synthesis of Hollow Co_3O_4 Microtube Arrays for Highly Efficient Water Electrolysis. *Angew. Chem.* **56**, 1324-1328 (2017).
23. Shi H, Liang H, Ming F, Wang Z. Efficient Overall Water‐Splitting Electrocatalysis Using Lepidocrocite VOOH Hollow Nanospheres. *Angew. Chem.* **56**, 573-577 (2017).
24. Yu LX, *et al.* Cu nanowires shelled with NiFe layered double hydroxide nanosheets as bifunctional electrocatalysts for overall water splitting. *Energ. Environ. Sci.* **10**, 1820-1827 (2017).
25. Zhang J, *et al.* Interface Engineering of MoS_2/Ni_3S_2 Heterostructures for Highly Enhanced Electrochemical Overall‐Water‐Splitting Activity. *Angew. Chem.* **55**, 6702-6707 (2016).
26. Chen G, *et al.* Efficient and Stable Bifunctional Electrocatalysts Ni/Ni_xM_y (M = P, S) for Overall Water Splitting. *Adv. Funct. Mater.* **26**, 3314-3323 (2016).
27. Costantino U, Marmottini F, Nocchetti M, Vivani R. New Synthetic Routes to Hydrotalcite-Like Compounds − Characterisation and Properties of the Obtained Materials. *Eur. J. Inor. Chem.* **1998**, 1439-1446 (1998).
28. Hibino T, Ohya H. Synthesis of crystalline layered double hydroxides: Precipitation by using urea hydrolysis and subsequent hydrothermal reactions in aqueous solutions. *Appl. Clay Sci.* **45**, 123-132 (2009).
29. Liu J, *et al.* Synthesis and thermal properties of ZnAl layered double hydroxide by urea hydrolysis. *Powder Technol.* **253**, 41-45 (2014).
30. Rao MM, Reddy BR, Jayalakshmi M, Jaya VS, Sridhar B. Hydrothermal synthesis of Mg–Al hydrotalcites by urea hydrolysis. *Mater. Res. Bull.* **40**, 347-359 (2005).
31. Zeng H, Deng X, Wang Y, Liao K. Preparation of Mg‐Al hydrotalcite by urea method and its catalytic activity for transesterification. *Aiche J.* **55**, 1229-1235 (2009).

32. Kang H, Leoni M, He H, Huang G, Yang X. Well‐Crystallized CO_3^{2-} Type LiAl‐LDH from Urea Hydrolysis of an Aqueous Chloride Solution. *Eur. J.Inor. Chem.* **2012**, 3859-3865 (2012).
33. Kloprogge JT, *et al.* Characterization of Intercalated Ni/Al Hydrotalcites Prepared by the Partial Decomposition of Urea. *Cryst. Growth Des.* **6**, 1533-1536 (2006).
34. Li Y, *et al.* Topochemical synthesis of Ni^{2+}–Fe^{3+} layered double hydroxides with large size. *Appl. Clay Sci.* **52**, 51-55 (2011).
